# Multilab EcoFAB study shows highly reproducible physiology and depletion of soil metabolites by a model grass

**DOI:** 10.1111/nph.15662

**Published:** 2019-01-24

**Authors:** Joelle Sasse, Josefine Kant, Benjamin J. Cole, Andrew P. Klein, Borjana Arsova, Pascal Schlaepfer, Jian Gao, Kyle Lewald, Kateryna Zhalnina, Suzanne Kosina, Benjamin P. Bowen, Daniel Treen, John Vogel, Axel Visel, Michelle Watt, Jeffery L. Dangl, Trent R. Northen

**Affiliations:** ^1^ Lawrence Berkeley National Laboratory 1 Cyclotron Road Berkeley CA 94720 USA; ^2^ Joint Genome Institute 2800 Mitchell Drive Walnut Creek CA 94598 USA; ^3^ Institut für Bio‐ & Geowissenschaften Forschungszentrum Jülich Wilhelm‐Johnen‐Straße 52428 Jülich Germany; ^4^ Department of Biology Howard Hughes Medical Institute University of North Carolina Chapel Hill 250 Bell Tower Drive Chapel Hill NC 27599 USA; ^5^ Institute of Molecular Plant Biology ETH Zürich Universitätsstrasse 2 8092 Zürich Switzerland; ^6^ School of Natural Sciences University of California Merced CA 95343 USA

**Keywords:** *Brachypodium distachyon*, metabolomics, model ecosystem, reproducibility study, rhizosphere processes, root exudates, root morphology, soil extract

## Abstract

- There is a dynamic reciprocity between plants and their environment: soil physiochemical properties influence plant morphology and metabolism, and root morphology and exudates shape the environment surrounding roots. Here, we investigate the reproducibility of plant trait changes in response to three growth environments.
- We utilized fabricated ecosystem (EcoFAB) devices to grow the model grass *Brachypodium distachyon* in three distinct media across four laboratories: phosphate‐sufficient and ‐deficient mineral media allowed assessment of the effects of phosphate starvation, and a complex, sterile soil extract represented a more natural environment with yet uncharacterized effects on plant growth and metabolism.
- Tissue weight and phosphate content, total root length, and root tissue and exudate metabolic profiles were consistent across laboratories and distinct between experimental treatments. Plants grown in soil extract were morphologically and metabolically distinct, with root hairs four times longer than with other growth conditions. Further, plants depleted half of the metabolites investigated from the soil extract.
- To interact with their environment, plants not only adapt morphology and release complex metabolite mixtures, but also selectively deplete a range of soil‐derived metabolites. The EcoFABs utilized here generated high interlaboratory reproducibility, demonstrating their value in standardized investigations of plant traits.

## Introduction

Plants adapt to their belowground environment by root morphological and metabolic plasticity. In turn, they influence soil physiochemical properties and root‐associated organisms by creating the rhizosphere, an environmental niche formed by the physical structure of roots and the release of metabolites (root exudates). These complex root–environment interactions are challenging to study in general, and even more so in a manner that is reproducible across laboratories (Poorter *et al*., 2012).

Root morphology and metabolism are affected by abiotic and biotic factors. Nutrient availability of soils, for example, can profoundly affect root morphology and provoke changes in root metabolism. Phosphate limitation typically results in elongated lateral roots and root hairs in a context‐dependent manner (Peret *et al*., 2011; Plaxton & Tran, 2011; Nestler *et al*., 2016) and in increased exudation of organic acids that solubilize phosphate (Neumann & Martinoia, 2002; Plaxton & Tran, 2011; Thijs *et al*., 2016). Root morphology and metabolism are further affected by microbes and microbial compounds (Venturi & Keel, 2016; Verbon & Liberman, 2016; Etalo *et al*., 2018). The presence of plant growth‐promoting bacteria can stimulate lateral root and root hair growth of Arabidopsis (López‐Bucio *et al*., 2007; Vacheron *et al*., 2013; Zamioudis *et al*., 2013). Plant responses to abiotic and biotic factors are likely intertwined, as illustrated recently by a study that linked phosphate stress in plants with the structure of root‐associated microbial communities (Castrillo *et al*., 2017). Thus, plant phenotypes in soil are a result of a complex response to abiotic and biotic factors, and an integrated view of root morphology and metabolism is necessary to gain a holistic understanding of plant–environment interactions.

Characterization of plant phenotypes in response to abiotic and biotic stresses in soil can have a profound impact on agriculture, especially as many resources, such as phosphate‐based fertilizers, are limited (Cordell *et al*., 2009), and global food demand is projected to have to increase by 60% by the year 2050 due to an ever‐growing population (FAO. World Food Situation http://www.fao.org/worldfoodsituation/en/ [accessed 15 May 2018]). Grasses are central to biofuel production and provide 70% of human calories (Brutnell *et al*., 2015). Thus, research on model grasses such as *Setaria viridis* and *Brachypodium distachyon* can inform growth strategies for many crops (Brutnell *et al*., 2015). *B. distachyon* is gaining popularity as a model grass because of its small genome, short generation time, genetic tractability, and the availability of extensive germplasm and mutant collections (Hsia *et al*., 2017). Additionally, since it uses C_3_ carbon (C) fixation, it is a good laboratory model plant relevant to cereal crops such as barley (*Hordeum vulgare*), rice (*Oryza sativa*), and wheat (*Triticum aestivum*). It has recently been utilized to investigate plant developmental processes, abiotic stresses, biotic interactions, and root morphology (Watt *et al*., 2009; Brutnell *et al*., 2015).

The relationship between plants and their environment is ideally studied in an agriculturally relevant field setting. Environmental factors, especially the type of soil in which plants are grown, are major determinants of root‐associated microbial communities (Bulgarelli *et al*., 2013; Edwards *et al*., 2015), and of root morphology (Senga *et al*., 2017). However, investigation of root morphology in soil is challenging due to its opacity, and investigation of exudation in soil is challenging due to soils physiochemical complexity (Cai *et al*., 2011). Specialized imaging techniques, such as magnetic resonance imaging, computed tomography (Metzner *et al*., 2015; Helliwell *et al*., 2017), or the use of labeled plants (Rellán‐Álvarez *et al*., 2015), have been developed, but they are not widely accessible or amenable to high‐throughput experimentation (Metzner *et al*., 2015). Similarly, approaches for the investigation of root exudation in soils include the use of *in situ* soil drainage systems (lysimeters) in fields (Strobel, 2001), which are low throughput and require complex installations, or of laboratory‐based extraction methods that are based on flushing the soil with large volumes of liquids (Swenson *et al*., 2015; Pétriacq *et al*., 2017). Studying metabolites within rhizosphere soils is also challenging because of the complex mixture of plant‐ and microbe‐derived metabolites, which are potentially altered by the chemistry and mineralogy of the soil investigated. A further challenge is the limited reproducibility of morphological and metabolic data generated (Massonnet *et al*., 2010; Poorter *et al*., 2012).

Owing to these challenges in the field, root morphology and metabolism are often studied in laboratory settings. Laboratory environments can feature transparent substrates and mineral growth media devoid of complex chemical compounds present in soils, in order to allow straightforward investigation of plant traits. However, these highly artificial laboratory environments may not reproduce normal plant growth and plant–environment interactions that occur in the field. Thus, systems that allow the manipulation of aspects of natural systems in a controlled laboratory environment are desirable. Microfluidic devices are gradually improved to study, for example, heterogenous environments (Stanley *et al*., 2017), and currently these important devices are designed to accommodate plants with small roots, such Arabidopsis, for a growth period of several days to *c*. 2 wk (Parashar & Pandey, 2011; Jiang *et al*., 2014; Stanley *et al*., 2017). We recently reported on a modular growth system, the EcoFAB (Ecosystem Fabrication), which facilitates the evaluation of root morphology and exudation of various plants over the course of several plant developmental stages up to several weeks (Gao *et al*., 2018). The EcoFAB design is purposely kept simple and inexpensive, to allow for straightforward design and manufacturing of EcoFABs for various experimental questions. The EcoFABs also address the challenge of studying plant growth in various environments, such as chemically simple or complex hydroponic setups, including the ability to add solid substrates such as sand or soil. In addition, microbes can be added to EcoFAB chambers, and the system is compatible with chemical imaging (Gao *et al*., 2018). One of the key distinctions of a standardized system such as the EcoFAB is the reproducibility of data generated.

The study presented here aimed to test the reproducibility of EcoFABs across multiple laboratories in assessing the response of the model grass *B. distachyon* in different growth media. Phosphate‐sufficient and ‐deficient mineral media were chosen to assess the performance of the EcoFAB system in reproducing well‐described effects of phosphate starvation, and a complex sterilized soil extract was chosen as representation of a more natural environment with yet uncharacterized effects on plant morphology and metabolism. We hypothesized that the use of the EcoFAB system produces data reproducible across laboratories, and that *B. distachyon* grown in the various media would result in distinct metabolic and morphological changes.

## Materials and Methods

### EcoFAB preparation

EcoFAB devices were fabricated according to the published method (Gao *et al*., 2018). Briefly, a 1 : 10 silicone elastomer curing agent : base mixture (polydimethylsiloxane (PDMS); Ellsworth Adhesives, Germantown, WI, USA) was poured onto a 3D‐printed mold, and allowed to solidify at 80°C for 4 h. The PDMS layer was separated from the mold, the edged trimmed, and permanently bonded to a glass microscope slide. The EcoFAB device and outer chamber were sterilized by incubation in 70% v/v ethanol for 30 min, followed by incubation in 100% v/v ethanol for 5 min. After evaporation of residual ethanol, the EcoFAB device was rinsed three times with the growth medium of choice before transferring seedlings.

### Plant growth conditions

All experiments were performed with *B. distachyon* Bd21‐3 (Vogel & Hill, 2007). Seeds were dehusked and sterilized in 70% v/v ethanol for 30 s, and in 6% v/v sodium hypochlorite, 0.1% v/v Triton X‐100 for 5 min, followed by five wash steps in water. Seedlings were germinated on 0.5× Murashige & Skoog (MS) plates (2.2 g l^−1^ MS medium, MSP01 (Caisson Labs, Smithfield, UT, USA) with 1650 mg l^−1^ ammonium nitrate, 6.2 mg l^−1^ boric acid, 332.2 mg l^−1^ calcium chloride, 0.025 mg l^−1^ cobalt chloride, 0.025 mg l^−1^ copper sulfate, 37.26 mg l^−1^ disodium EDTA, 27.8 mg l^−1^ ferrous sulfate heptahydrate, 180.7 mg l^−1^ magnesium sulfate, 16.9 mg l^−1^ manganese(II) sulfate monohydrate, 0.25 mg l^−1^ sodium molybdate dihydrate, 0.83 mg l^−1^ potassium iodide, 1900 mg l^−1^ potassium nitrate, 170 mg l^−1^ monopotassium phosphate (KH_2_PO_4_), 8.6 mg l^−1^ zinc sulfate heptahydrate; 6% w/v Bioworld phytoagar, 401000721 (Fisher Scientific, Waltham, MA, USA), pH adjusted to 5.7) in a 16 h : 8 h, light : dark regime at 24°C. EcoFABs were sterilized as published, and seedlings transferred to EcoFAB chambers at 3 d after germination as previously described (Gao *et al*., 2018). Seedlings with comparable size were picked to conduct the experiment and were distributed in a random manner to the various EcoFABs. EcoFABs were incubated in a 16 h : 8 h, light : dark regime at 24°C, with 150 μmol m^−2^ s^−1^illumination. The EcoFABs were filled with 2 ml of 0.5× MS (*B. distachyon* grows without phenotypically detectable nutrient limitation, ‘phosphate‐sufficient’, 2.2 g l^−1^ MS medium, MSP01 (Caisson Labs), pH adjusted to 5.7), 0.5× MS‐P (*B. distachyon* leaves turn yellow as a sign of malnutrition, ‘phosphate‐deficient’, 2.2 g l^−1^ MS medium without phosphate, composition is the same as MSP01 without 170 mg l^−1^ KH_2_PO_4_, MSP11 (Caisson Labs), pH adjusted to 5.7), or soil extract. The soil extract was prepared by incubating 100 g of a standard glasshouse soil (Pro‐Mix PGX; Hummert International, Earth City, MO, USA) in 1 l of water for 16 h at 4°C and gentle shaking, followed by filtration through a 0.2 μm cellulose nitrate filter (09‐761‐104; Corning, Tewksbury, MA, USA) for sterilization. The soil extract was stored at 4°C, and its phosphate content was determined as 145 μM (Ames, 1966), which is four times lower than 0.5× MS. Although we did not perform additional nutrient analyses, it is likely that levels of other nutrients besides phosphate are also low, compared with 0.5× MS.

A comparative study of *B. distachyon* in EcoFABs vs plates was performed by laboratory 1, in which *B. distachyon* seeds were sterilized and germinated on 0.5× MS plates for 3 d as already described, then either transferred to EcoFAB growth chambers containing 0.5× MS liquid medium as described (Gao *et al*., 2018) or to 0.5× MS phytoagar plates. Roots were imaged weekly, and total root area was measured using the imageJ software suite (v.2.0.0). For the developmental time course, plants were grown in EcoFAB chambers in 0.5× MS for up to 43 d, and exudates were collected at indicated times (Supporting information Fig. S1), frozen, and stored at −80°C. Metabolites were extracted as described in the section ‘Liquid chromatography‐mass spectrometry sample extraction’.

### EcoFAB interlaboratory experiment

**Figure 1:**
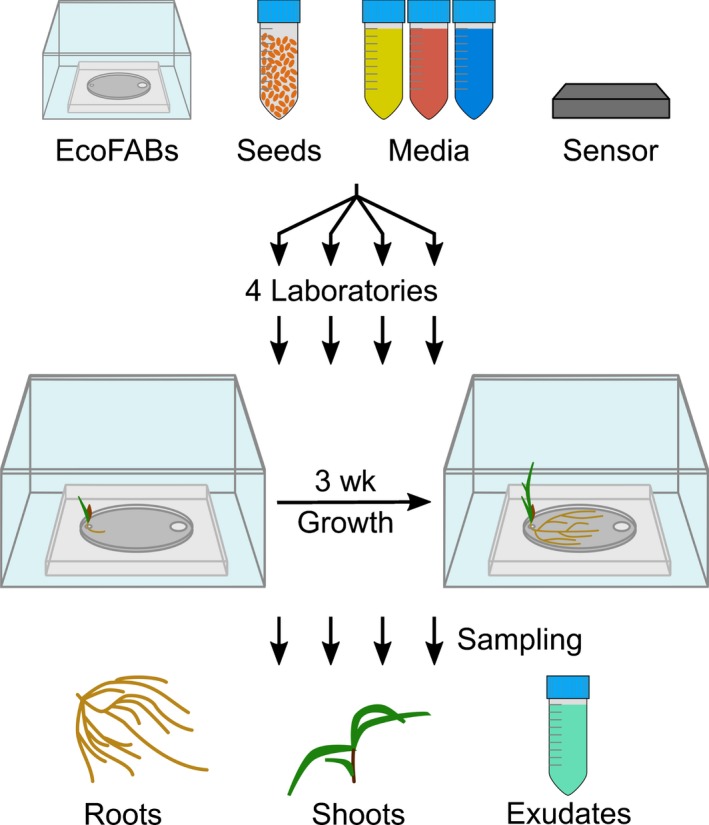
Experimental setup of the reproducibility experiment. Illustration of the reproducibility experiment: EcoFABs, *Brachypodium distachyon* seeds, growth media (yellow, 0.5× Murashige & Skoog (MS); red, 0.5× MS‐P; blue, soil extract), and light/temperature sensors were distributed to the participating laboratories. Each laboratory germinated the seeds, transferred seedlings to sterilized EcoFABs, and grew the plants for 21 d. Root and shoot tissue as well as root exudates were sampled for downstream analysis.

An overview of the experimental procedure is provided in Fig. 1, and the participating laboratories are listed in Table S1. The following material was distributed from laboratory 1 to the participating laboratories: EcoFAB growth chambers, micropore tape to seal the EcoFABs, *B. distachyon* seeds, MS powder, MS‐P powder, liquid soil extract (see Plant growth conditions section), phytoagar, and light and temperature data loggers (UA‐002‐08; HOBO Onset, Bourne, MA, USA), and a detailed protocol for plant growth and experimental procedures. The experiments were conducted in parallel by the different laboratories. Each participating laboratory sterilized EcoFABs and seeds as described (Gao *et al*., 2018). Growth conditions were monitored throughout the experiment, and these are reported in Table S1. Plants were grown in quadruplicates for each experimental condition, and one control EcoFAB was set up per condition without plants. Sterility was monitored throughout the experiment by plating 50 μl of growth media on Luria–Bertani plates every week. Of the 60 chambers total, four chambers were excluded from analysis due to contamination.

Root systems in EcoFABs were imaged at 7, 14, and 21 d after transfer (dat) to the EcoFAB chambers. Total root length was quantified by laboratory 1 with the smartroot plugin (v.4.21) for the imagej software (v.2.0.0) (Lobet *et al*., 2011). Root hairs were imaged at 21 d with 10× magnification, and their length was determined with imagej. The data presented are an average of three measurements per imaged root.

Growth media were replenished to 2 ml three times a week, and the media were exchanged fully at 20 dat. This medium was removed through the sampling port by pipetting after 24 h of further incubation, and the volume was recorded. The root exudates were frozen immediately, stored at −80°C, and shipped to laboratory 1 for metabolite analysis. The FW of root and shoot tissue was recorded, and the tissue was immediately frozen and stored at −80°C. The tissue was homogenized by the participating laboratories by their method of choice (mortar and pestle with liquid nitrogen (N), or steel beads with a bead beater). An aliquot of the tissue was utilized for phosphate content determination by all participating laboratories (Ames, 1966), and an aliquot was sent back to laboratory 1 for metabolite analysis.

### Liquid chromatography–mass spectrometry sample extraction

Homogenized root tissues were extracted two times with 700 μl 100% liquid chromatography–mass spectrometry (LC–MS)‐grade methanol (CAS 67‐56‐1; Honeywell Burdick & Jackson, Morristown, NJ, USA) for 1 h at 4°C. The samples were centrifuged for 5 min at 5000 ***g***, 4°C, and supernatants were pooled and evaporated under vacuum at 25°C until dry. The samples were resuspended in 100% LC–MS‐grade methanol with 15 μM internal standards (767964; Sigma‐Aldrich) with a volume relative to the sample FW (11 mg per 100 μl).

Frozen root exudates were lyophilized using a Labconco FreeZone lyophilizer, resuspended in 500 μl LC–MS‐grade methanol (CAS 67‐56‐1; Honeywell Burdick & Jackson), sonicated for 15 min in a water bath at 23°C, and incubated at 4°C for 16 h for salt precipitation. Samples were then centrifuged for 5 min at 5000 ***g***, 4°C, and supernatants were transferred to new microcentrifuge tubes and evaporated at 25°C under vacuum until dry. Samples were resuspended in 100% LC–MS‐grade methanol with 15 μM internal standards (767964; Sigma‐Aldrich) with a volume relative to the root tissue FW, and the root exudate volume collected (20 μl methanol 100 mg^−1^ FW per milliliter of exudate volume).

### LC–MS method and analysis

Metabolites in samples were chromatographically separated using hydrophilic liquid interaction chromatography on a SeQuant 5 μm, 150 mm × 2.1 mm, 200 Å zic‐HILIC column (1.50454.0001; Millipore) and detected with a Q Exactive Hybrid Quadrupole‐Orbitrap mass spectrometer equipped with an HESI‐II source probe (ThermoFisher Scientific, Waltham, MA, USA). For chromatographic separations, an Agilent 1290 series high‐performance LC system was used with a column temperature of 40°C, 3 μl sample injections, and 4°C sample storage. A gradient of mobile phase A (5 mM ammonium acetate in water) and B (5 mM ammonium acetate, 95% v/v acetonitrile in water) was used for metabolite retention and elution as follows: column equilibration at 0.45 ml min^−1^ in 100% B for 1.5 min, a linear gradient at 0.45 ml min^−1^ to 35% A over 13.5 min, a linear gradient to 0.6 ml min^−1^ and to 100% A over 3 min, a hold at 0.6 ml min^−1^ and 100% A for 5 min followed by a linear gradient to 0.45 ml min^−1^ and 100% B over 2 min and re‐equilibration for an additional 7 min. Each sample was injected twice: once for analysis in positive‐ion mode and once for analysis in negative‐ion mode. The mass spectrometer source was set with a sheath gas flow of 55, aux gas flow of 20 and sweep gas flow of 2 (arbitrary units), spray voltage of |±3| kV, and capillary temperature of 400°C. Ion detection was performed using the Q Exactive's data dependent MS2 Top2 method, with the two highest abundance precursory ions (2.0 *m*/*z* isolation window, 17 500 resolution, 1 × 10^5^ automatic gain control (AGC) target, 2.0 *m*/*z* isolation window, stepped normalized collisions energies of 10, 20, and 30 eV) selected from a full MS pre‐scan (70–1050 *m*/*z*, 70 000 resolution, 3 × 10^6^ AGC target, 100 ms maximum ion transmission) with settings at 1 × 10^3^ minimum AGC target, charges excluded above |3| and a 10 s dynamic exclusion window. Internal and external standards were included for quality control purposes, with blank injections between every unique sample.

### Metabolite identification and statistical analysis

LC–MS data were analyzed with Metabolite Atlas to construct extracted ion chromatograms corresponding to metabolites contained within our in‐house standards library (https://github.com/biorack/metatlas; Bowen & Northen, 2010; Yao *et al*., 2015). For metabolite identification, chemical classes were assigned using the classyfire compound classification system (Djoumbou Feunang *et al*., 2016). Metabolites were identified following the conventions defined by the Metabolomics Standards Initiative (Sumner *et al*., 2007; Tables S2, S3). All assignments were of the highest confidence (‘level 1’ Metabolomics Standards Initiative identifications), which is identified as at least two orthogonal measures vs authentic chemical standards (e.g. retention time and fragmentation spectra). In all cases we used three orthogonal measures: retention time (within 1 min vs standard), fragmentation spectra (manual inspection), and accurate mass (within 20 ppm). In general, accurate masses were within 5 ppm, though the error was higher for low‐mass ions in negative mode. Peak height and retention time consistency for the LC–MS run were ascertained by analyzing quality control samples that were included at the beginning, during, and at the end of the run. Internal standards were used to assess sample‐to‐sample consistency for peak area and retention times.

Metabolite background signals detected in the extraction blanks, 0.5× MS, and 0.5× MS‐P control samples were subtracted from the experimental sample peak heights. Further, metabolite peak heights were normalized by setting the maximum peak height detected in any sample to 100%. The method utilized here allows for the relative comparison of peak heights between samples (e.g. if a compound of interest is present in significantly different amounts between samples), but not for absolute metabolite level quantification (e.g. micrograms of a compound of interest per gram tissue). To explore the variation between growth conditions, the metabolite profiles were principal component analysis ordinated, and the 95% confidence level was displayed as ellipses for each treatment. Hierarchical clustering analysis with a Bray–Curtis dissimilarity matrix was performed with the Python 2.7 Seaborn package. The significance between root tissue as well as root exudate metabolic profiles was analyzed with the Python SciPy ANOVA test coupled to a Python Tukey's honestly significant difference test with *α* = 0.05 corresponding to a 95% confidence level for each metabolite. Statistically significant metabolites were displayed as bar graphs, where the sum of all values added up to 100% (Figs 4 (see later), S2), or as fold change for soil extract exudates divided by soil extract controls (see Fig. 5).

## Results

### The EcoFAB growth system design and benchmarking

The EcoFAB device is comprised of a PDMS layer bonded to a glass slide, an outer box to maintain sterility (Gao *et al*., 2018), a plant reservoir to hold the seedling, and a sampling port for the addition or exchange of growth medium (Fig. S1a). *Brachypodium distachyon* can be grown in EcoFABs for multiple weeks (Fig. S1b depicts a 3‐wk‐old *B. distachyon* plant), facilitating the investigation of various developmental stages from seedlings to adult plants.

We benchmarked *B. distachyon* growth in the EcoFAB vs on standard agar plates. We found that *B. distachyon* roots develop similarly in EcoFABs containing 0.5× MS medium compared with growth on 0.5× MS agar plates over the course of 5 wk, with no significant differences in total root area observed except for week 2 (*P* = 0.05; Fig. S1c). In addition, sampling of *B. distachyon* root exudates at different developmental stages showed a gradual shift of exudate profiles over time (Fig. S1d), consistent with reports for plants in other growth systems (Chaparro *et al*., 2013; Zhalnina *et al*., 2018).

### Multilab investigation of EcoFAB data reproducibility

EcoFab materials were distributed to four participating laboratories that ran the same experiment in parallel, investigating morphological and metabolic changes of *B. distachyon* grown in phosphate‐sufficient, phosphate‐deficient, or soil extract medium (4.3 times less phosphate than phosphate‐sufficient medium). Roots were imaged on a weekly basis, and after 3 wk each laboratory determined the FW and phosphate content of root and shoot tissue, and sampled root tissue and exudates for LC–MS analysis (Fig. 1).

**Figure 2:**
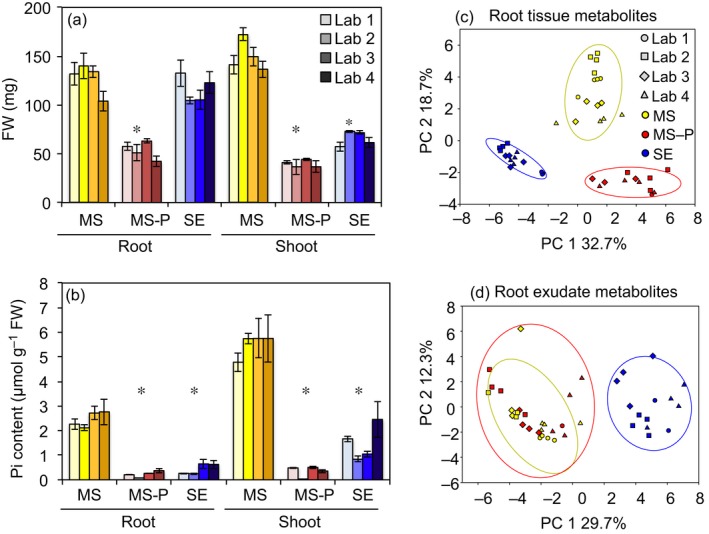
Interlaboratory morphological and metabolic consistency. *Brachypodium distachyon* was grown in 0.5× Murashige & Skoog (MS) (MS, yellow), 0.5× MS‐P (MS‐P, red), or soil extract (SE, blue) for 3 wk. Root and shoot (a) FW and (b) phosphate content were determined by the participating laboratories. Data are means ± standard error (*n *>* *9). Asterisks indicate significant differences between experimental treatments (ANOVA, *P *<* *0.05). Principal component (PC) analysis of normalized peak heights of (c) ground root tissue metabolites and (d) root exudate metabolites. Hierarchical clustering for the metabolite data is shown in Supporting Information Fig. S4.

Growth conditions (light intensity, day length, and temperature) were comparable between laboratories throughout the experiment (Table S1). The FW and phosphate content were consistent across laboratories, and different between experimental treatments (Fig. 2a,b): as expected, phosphate‐deficient plants had significantly lower phosphate content, and less than half the FW of phosphate‐sufficient plants (Tukey's test, *P* = 0.05). Interestingly, soil‐extract‐grown plants showed a mixed response, in that they resembled phosphate‐deficient plants in phosphate content and shoot weight, but their root weight was significantly higher than of phosphate‐deficient plants, and more similar to phosphate‐sufficient plants. The root : shoot FW ratio averaged across all laboratories was 0.9 for phosphate‐sufficient plants, 1.3 for phosphate‐deficient plants, and 1.8 for soil‐extract‐grown plants (Fig. S3).

Upon receiving samples from each laboratory following the experiment, laboratory 1 extracted and analyzed metabolites, generating metabolite profiles from root tissues and exudates using LC–MS. The metabolic profiles of root tissues were comparable across laboratories, and reproducibly demonstrated a clear separation between experimental conditions in a principal component analysis plot and in a hierarchical clustering analysis (Figs 2c, S4a; Tukey’ honestly significance test, *P* = 0.05). Similarly, the metabolic profiles of root exudates were comparable across laboratories and showed a separation between soil extract and other growth conditions (Figs 2d, S4b).

**Figure 3:**
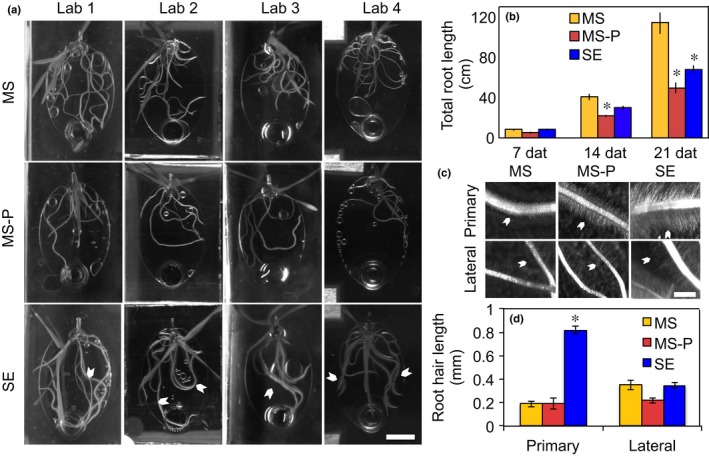
Root morphology. (a) Representative pictures of 14 d after transfer (dat) *Brachypodium distachyon* in EcoFAB chambers in 0.5× Murashige & Skoog (MS) (MS), 0.5× MS‐P (MS‐P), or soil extract (SE) for the different laboratories (Lab 1–Lab 4). Note the long root hairs in soil‐extract growing plants (arrowheads). Brightness and contrast were adjusted for better display. (b) Total root length 7, 14, and 21 dat averaged across laboratories. The same data are displayed per lab in Supporting Information Fig. S2. Data are means ± standard error (*n *>* *9). (c) Root hair morphology. Arrowheads point to root hairs. (d) Root hair length at 21 dat for primary and lateral roots. Data are means ± standard error (*n *>* *9). Asterisks indicate significant differences within a group of bars (ANOVA, *P *<* *0.05). Bars: (a) 1 cm; (c) 1 mm.

Root morphology (quantified by laboratory 1) was similarly different between experimental treatments. Plants grown in phosphate‐sufficient conditions formed root systems extending across most of the EcoFAB root chambers, whereas phosphate‐deficient roots did not reach as far. Soil‐extract‐grown roots also reached across the entire root chamber, with overall less roots compared with phosphate‐sufficient plants, but visibly elongated root hairs (Fig. 3a). Quantification of total root length averaged across laboratories was 7 cm at 7 dat for all plants, increased to 40 cm, 22 cm, and 30 cm at 14 dat, and further to 114 cm, 48 cm, and 67 cm for phosphate‐sufficient, phosphate‐deficient, and soil‐extract‐grown plants, respectively. Differences between experimental treatments were first visible 14 dat with phosphate‐deficient plants exhibiting shorter total root length than phosphate‐sufficient plants (Tukey, *P* = 0.05), but became more pronounced by 21 dat, with phosphate‐sufficient plants exhibiting longer total root length than those grown in soil extract, which in turn were longer than of phosphate‐deficient plants (Fig. 3b). Interestingly, root morphology varied somewhat between laboratories: the absolute measurements differed up to a factor of 2, with plants grown in laboratories 1 and 4 exhibiting consistently higher total root length than plants of laboratories 2 and 3 (Fig. S2). Specifically, total root length was 75–150 cm in phosphate‐sufficient, 32–62 cm in phosphate‐deficient, and 44–87 cm in soil‐extract conditions (Fig. S2).

To summarize, the root and shoot FW and phosphate content, root and exudate metabolic profiles, and total root length were consistent across laboratories and distinct for the experimental treatments.

### Distinct root morphology in soil extract

In addition to the high root : shoot ratio observed for soil‐extract‐grown plants (Fig. S3), plants grown in soil extract had longer root hairs than plants grown in other conditions, which were visible even under low‐magnification (Fig. 3a,c). Interestingly, quantification revealed that root hairs on primary soil‐extract‐grown roots reached a length of 0.8 mm, which was four times longer than phosphate‐sufficient‐ or phosphate‐deficient‐grown roots. Root hair length of lateral roots remained unchanged (Fig. 3d).

### Metabolic analysis of root tissue and exudates

Metabolites extracted from root tissue and root exudates were found to be distinct between experimental treatments (Fig. 2c,d). Based on authentic metabolite standards, a broad range of metabolites was detected in root tissues as well as in exudates; among them were organic acids, carbohydrates, nucleosides/nucleotides/nucleic bases, amino acids and other nitrogenous compounds, benzenoids, and fatty acids.

**Figure 4:**
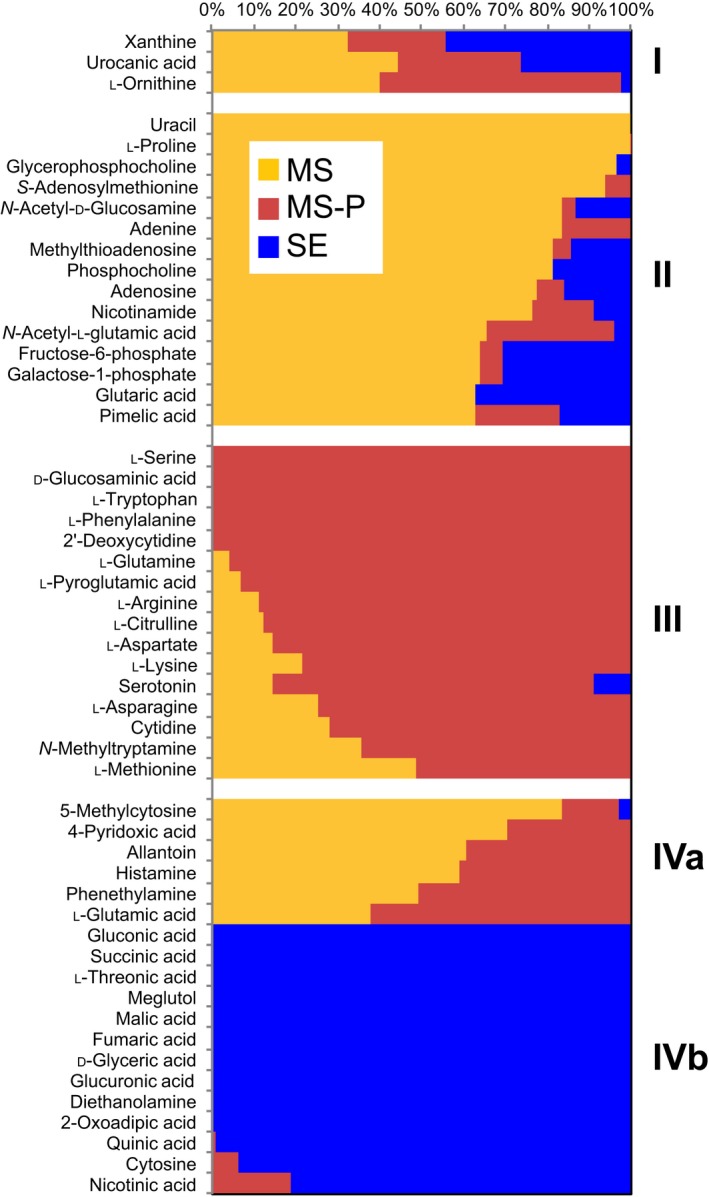
Characteristic metabolites detected in different root tissues. Normalized relative peak height of metabolites differing between roots grown in 0.5× Murashige & Skoog (MS) (MS, yellow), 0.5× MS‐P (MS‐P, red), and soil extract (SE, blue) (ANOVA, *P *<* *0.05). Metabolite clusters are indicated by roman numerals.

Half of the metabolites detected in root tissue extracts (52 out of 117 compounds) were significantly different in pairwise comparisons of experimental treatments, with 28% having highest abundance in phosphate‐sufficient‐, 30% in phosphate‐deficient‐, and 25% in soil‐extract‐grown roots (Fig. 4; Table S2). The significantly different metabolites (*P* < 0.05) could be grouped into four main clusters (Fig. 4). Cluster I consists of three metabolites significantly different between all experimental treatments. Cluster II is composed of metabolites abundant in phosphate‐sufficient roots, including nucleosides, organic acids, amino acids, and, notably, all phosphorous compounds present in this dataset. The higher abundance of phosphorous compounds in phosphate‐sufficient roots compared with phosphate‐deficient‐ or soil‐extract‐grown roots is in line with the phosphate quantification of plant tissues (Fig. 2b), in which the highest free phosphate was detected in phosphate‐sufficient plants, as would be expected. Cluster III includes metabolites abundant in phosphate‐deficient roots. All these metabolites are nitrogenous compounds, likely due to the N–phosphate imbalance of phosphate‐deficient plants. Cluster IV contains metabolites distinct for soil‐extract‐grown roots, and is split in two subclusters: IVa includes metabolites with low abundance in soil extract roots, which are mostly nitrogenous compounds, whereas IVb includes metabolites with high abundance in soil extract roots, which are mostly organic acids.

Overall, 137 metabolites were identified in root exudates (Table S3). Only phenylacetaldehyde was significantly different between exudates of phosphate‐sufficient and ‐deficient plants (Table S3), which explains why these conditions are not separated in a principal component analysis (Fig. 2d). Plants grown in soil extracts had a distinct exudate composition, with 27 and 25 distinct compounds vs phosphate‐sufficient and phosphate‐deficient root exudates, respectively. Most of these distinct metabolites were most abundant in soil extract controls (no plant), showed medium abundance in soil extract exudates, and had low abundance in the other conditions (Fig. S2).

**Figure 5:**
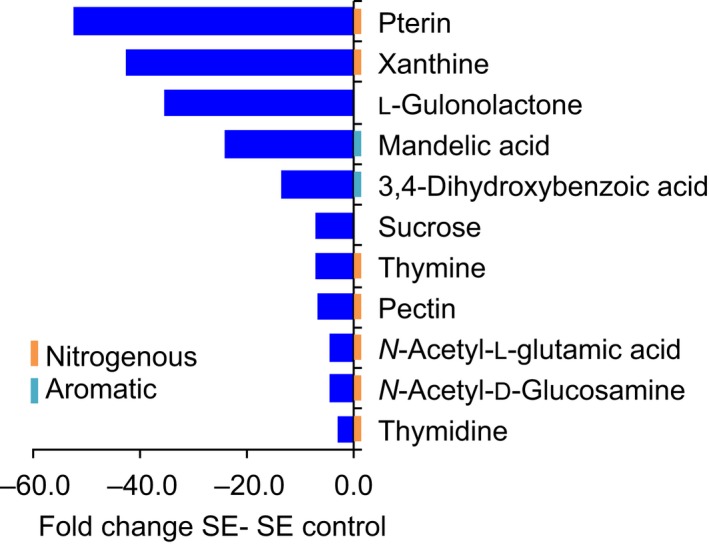
Metabolites reduced in exudates of soil‐extract‐grown plants. Fold change of selected metabolites differing between exudates of plants grown in soil extract (SE), and soil extract controls (ANOVA, *P *<* *0.05). Graphs for single laboratories are given in Supporting Information Fig. S7.

Metabolite comparisons between soil extract with and without plants revealed that half of the metabolites detected (74 of 136 compounds) were altered in abundance, causing a distinct grouping in a principal component analysis (Fig. S5). Fifty percent of these metabolites were depleted in the presence of plants (Table S3; Fig. S6). Although individual metabolite levels varied somewhat across laboratories, this finding was consistent across participating laboratories (Fig. S7). Distinct metabolites included organic acids, carbohydrates, amino acids, and nucleosides, and these compounds contained various groups, such as phosphate, N, or sulfur (S) (Fig. 5; Table S3). Furthermore, citric acid exhibited an interesting but statistically insignificant trend of higher abundance in soil extract exudates vs controls (Table S3; ANOVA, *P* = 0.23; *t*‐test, *P* = 0.04).

Metabolites that were detected in root tissue and root exudates showed distinct patterns: 42% of these metabolites were significantly different in roots and 43% in exudates, depending on environments. Only 23% of the compounds were significantly different in both datasets, which indicates that root exudates are metabolically distinct from root tissue (Figs 4, S6; Tables S2, S3). We similarly found that only 50% of the metabolites depleted from soil extract were significantly different in root tissues, with 29% of high abundance in soil extract roots (mostly organic acids), 25% of low abundance, and 46% are not detected (nitrogenous compounds).

## Discussion

### Reproducibility of morphological and metabolic data in EcoFABs

This study investigated the reproducibility of morphological and metabolic responses of the model grass *B. distachyon* grown in EcoFABs in phosphate‐sufficient and phosphate‐deficient mineral medium and in chemically complex but sterile soil extract. We purposely chose phosphate starvation as an experimental system, as the morphological and metabolic responses of plants are well described and should be reproducible in a system such as the EcoFAB. The soil extract medium was added to represent a more natural environment, but it was sterilized to exclude the effects of microbial metabolism on exudation and to lower variability of the system.

We found that *B. distachyon* FW, phosphate content, and metabolic profiles were distinct for our experimental conditions and that these responses were reproducible across the four participating laboratories. The traits investigated included tissue FW and phosphate content, total root length, and metabolic profiles of roots and exudates. These results compare favorably to a related study comparing three A*rabidopsis thaliana* genotypes grown in soil in pots by 10 laboratories (Massonnet *et al*., 2010) where, similar to this study, materials were distributed from one laboratory, growth conditions were monitored at each laboratory, and one laboratory analyzed leaf morphology and metabolomic and transcriptomic profiles. Although one trait was similar between a core group of four laboratories, all traits significantly varied across laboratories. The authors attributed the variance to the strong influence of small environmental changes in their soil pot system (Massonnet *et al*., 2010). Our EcoFAB setup comprised a more uniform and controlled growth environment than pots filled with soil, which is likely one cause of the higher reproducibility observed here. Another equalizing factor might have been the use of sterilized soil extract in this study, which did not take into consideration the complex physical and mineral properties of soil, or the effects of microorganisms. It could be that integrating these factors in future EcoFAB studies might increase the variability of the system. It will be important to investigate the reproducibility as well as the morphological and metabolic responses of plants to microbial communities and soil mineralogy, as natural soils were identified as main contributors shaping root morphology, plant C exudation, plant–microbe interactions, and rhizosphere extension (Bulgarelli *et al*., 2013; Holz *et al*., 2017; Koebernick *et al*., 2017; Edwards *et al*., 2018). Overall, we conclude that the reproducibility of plant traits in soil extract EcoFABs is a promising first step towards developing plant growth systems generating reproducible data that are relevant to field environments.

### Metabolic profiles of roots were more distinct than of exudates

Root metabolic profiles were clearly distinct between experimental treatments. Phosphate‐sufficient roots were abundant in nucleosides, amino acids, organic acids, and phosphorous compounds, whereas phosphate‐deficient roots accumulated nitrogenous compounds, and soil‐extract‐grown roots were deficient in nitrogenous compounds, but accumulated carbohydrates (Fig. 4). It will be interesting to investigate whether shoot metabolic profiles are similarly distinct between experimental treatments in a future study.

The metabolites detected in *B. distachyon* root exudates in this study (Table S3) were comparable to metabolites detected in exudates of other grasses, such as wheat (Iannucci *et al*., 2017), maize (*Zea mays*; Carvalhais *et al*., 2011), rice (Bacilio‐Jiménez *et al*., 2003), *Avena barbata* (Zhalnina *et al*., 2018), and dicots such as Arabidopsis (Chaparro *et al*., 2013). Similarly, the *B. distachyon* exudation profile varied with developmental stage, as reported for other plants (Fig. S1; Chaparro *et al*., 2013; Zhalnina *et al*., 2018).

The largest exudate metabolic differences in this study were observed between plants grown in soil extract and soil extract controls without plants. Surprisingly, we did not find many statistical differences in exudates of plants grown in phosphate‐sufficient vs ‐deficient conditions. For many plants, an increase in organic acid exudation in low phosphate conditions was reported (Neumann & Martinoia, 2002; Plaxton & Tran, 2011; Thijs *et al*., 2016), which was not found in our dataset. This might be due to several reasons. First, plants were grown without phosphate for the entire growth period and might have ceased differential exudation when sampled after 3 wk. Second, the small EcoFAB volume likely allows for re‐uptake of exuded metabolites, masking differential exudation of compounds. Third, the exudation response of *B. distachyon* to phosphate starvation might not be as pronounced as in other species and be below the detection limit in our assay. Future experiments focusing on the timing and magnitude of *B. distachyon* exudation changes in response to phosphate starvation would be able to address these points. The clear differences observed for FW, tissue phosphate content, and root metabolic profile indicate that the plants indeed were starved for phosphate in our experimental setup.

### Plants deplete metabolites from soil extract

The main differences in exudate metabolic profiles in this study were due to a depletion of metabolites from soil extract by plants (Fig. 5; Table S3). With our experimental setup, we are unable to determine whether metabolites are depleted due to uptake by plant roots or due to, for example, chemical reactions caused by an altered pH around plant roots. Experiments with isotopically labeled compounds spiked into soil extract could address the fate of metabolites of interest in future experiments.

In addition to depletion of metabolites, a trend for increased citric acid levels in soil‐extract‐grown plants was observed. This might constitute a starvation response, given that exudation of organic acids is a characteristic of phosphate‐limited plants (Neumann & Martinoia, 2002; Plaxton & Tran, 2011). The fact that half of the soil extract metabolites (among them, organic acids, amino acids, nucleosides, and carbohydrates) are depleted by plants is surprising, as it suggests that plants not only are producers, but also consumers of a significant amount of compounds. Various nitrogenous compounds are depleted from soil extract by plants. Among them is pterin, which is a folate precursor. Folate is an essential part of human diet, and thus studying uptake of pterin by plants to elevate folate levels might be an interesting biofortification strategy (Strobbe & Van Der Straeten, 2017). Xanthine is part of the purine degradation pathway in plants and can act as a sole N source for *A.  thaliana* growth (Brychkova *et al*., 2008). Similarly, there could be direct utilization of thymine and thymidine for synthesis of nucleic acids and of *N*‐acetyl‐l‐glutamic acid for synthesis of amino acids. In addition, plants deplete complex organoheterocyclic compounds such as the ascorbic acid precursor gulonolactone (Smirnoff, 2018), as well as simple carbohydrates such as sucrose. Uptake of these compounds by roots would indicate that plants grow partially heterotrophically in specific environments, importing simple and complex biomass precursors.

There is only a small amount of literature regarding uptake of metabolites by roots: amino acids and sugars were reported to be imported by roots in mineral medium assays where compounds were spiked in (Jones & Darrah, 1994; Yamada *et al*., 2011), whereas organic acids are likely not imported at significant amounts (Jones & Darrah, 1995). There is evidence that plants are capable of (re)importing C from environments (Jones & Darrah, 1993), but overall, the scope of how much and which metabolites are taken up by plants from natural environments is currently unknown. In another experimental system comprising cyanobacteria and associated heterotrophs, it was found that the primary producer depleted 26% of biological soil crust metabolites, whereas soil heterotrophs only depleted 13% of metabolites (Baran *et al*., 2015). This might suggest that photoautotroph organisms in general not only release, but also deplete, a significant amount of compounds from the environment. Plants might compete with microbes for nutrient soil organic compounds in certain environmental conditions. Besides nutritional functions, compounds could act as signals, as exemplified by a recent study that found the depletion of plant‐derived phenolic acids to be associated with rhizosphere microbes (Zhalnina *et al*., 2018).

Many of the plant‐depleted metabolites contained N, phosphate, or S groups (Fig. 5; Table S3), which suggests that plants not only use inorganic forms, but also more complex compounds as nutrients. Consistent with this hypothesis, compounds containing the N, phosphorus (P), and S groups are low in soil‐extract‐grown roots, likely indicating a fast turnover rate. It was suggested that amino acid uptake might account for 30–90% of imported N, depending on the environmental conditions (Jones & Darrah, 1994; Yamada *et al*., 2011), but overall, data on how much elements are taken up as inorganic vs organic compounds is missing. By contrast to N‐, P‐, and S‐containing compounds, carbohydrate‐type compounds were of high abundance in soil‐extract‐grown roots, likely due to a low external demand for carbohydrates by plant tissues (Fig. 4).

Interestingly, plants depleted metabolites from soil extract in a selective manner, suggesting that the plant controls depletion of metabolites to a certain degree. Similarly, the difference between root and exudate metabolic profiles (Figs 4, S6) indicates that plants control exudation to some degree. Selectivity in import and export processes could be achieved by the presence of transport proteins that were described for a number of metabolites (Sasse *et al*., 2018), and investigation of transport processes is a promising direction for future studies. We conclude that plants not only significantly alter their environment by export, but also by depletion of metabolites.

### Distinct plant growth in soil extract

In this study, plants were grown in basal salt medium widely used in standard laboratory settings, and in soil extract medium that includes water‐soluble metabolites but that excludes additional factors defining soils, such as presence of other metabolically active organisms or solid soil particles.

We observed an increased root : shoot ratio in plants grown in soil extract, which might point to nutrient limitations (Cai *et al*., 2011), consistent with the low phosphate content of soil extract and of soil‐extract‐grown plants (Figs 2b, S3). Interestingly, altered root : shoot ratios were recently also detected for wheat genotypes grown in different soils (Iannucci *et al*., 2017), suggesting that different soils might affect root : shoot ratio and possibly also metabolic profiles in different ways.

The most prominent phenotypic difference observed for soil‐extract‐grown plants was the four‐fold increase in root hair length compared with other plants (Fig. 3). Root hair elongation can be caused by altered nutrient levels (e.g. phosphate, N, potassium, iron, micronutrients) (Senga *et al*., 2017; Zhang *et al*., 2018), and depends on the growth condition used (Nestler *et al*., 2016). Further, the response to phosphate is concentration dependent (Bates & Lynch, 1996), which might be the cause for the different root hair phenotype observed in phosphate‐deficient medium vs phosphate‐limited soil extract. Alternatively, the presence of microbes and microbe‐derived metabolites that alter plant hormone homeostasis could also cause the phenotype observed in soil extract (López‐Bucio *et al*., 2007; Ortiz‐Castro *et al*., 2011; Vacheron *et al*., 2013; Zamioudis *et al*., 2013). Compounds such as tryptophan and salicylate detected in soil extract are reported to alter root morphology (Vacheron *et al*., 2013), and thus are candidates for causing elongated root hairs. We suggest that the long root hair phenotype observed could be a result of soil extract nutrient levels and specific concentrations of signaling compounds. The determination of the causal factor(s) resulting in the long root hair phenotype represents an important future direction.

Root hair length was shown to have a significant impact on how plants grow in natural soils, and how plants interact with their environment. Root hairs alter physical properties of the soil, such as the extension of the rhizosphere and the pore size development in soils (Holz *et al*., 2017; Koebernick *et al*., 2017). Root hairs also affect biotic interactions by defining the rhizosphere and the amount of C exuded from roots (Holz *et al*., 2017; Koebernick *et al*., 2017). The complex morphological and metabolic alterations of *B. distachyon* when grown in soil extract stress the importance of not only considering standard laboratory growth media, but also more natural substrates when studying plant–environment interactions. It would be interesting to investigate how root hair length changes when solid particles, microbial communities, or both are added back to the soil extract used in this study, to investigate morphology changes in a more natural environment. In addition, the observation that increased root hair length was restricted to primary roots but not observed on lateral roots highlights the need for high spatial resolution when measuring root traits, even in a simplified system like the EcoFAB.

In conclusion, EcoFABs are reproducible tools to study a variety of topics, and this reproducibility enables interlaboratory studies of plant–environment interactions. Their low cost, flexibility, and compatibility with metabolomics studies enables investigations of increasingly complex conditions simulating specific natural environments. We found that *B. distachyon* growth in EcoFABs was reproducible across four laboratories for a number of morphological and metabolic traits, including tissue FW and phosphate content, total root length, and metabolic profiles of root tissue and root exudates. In addition, plants grown in soil extract exhibited an altered root : shoot ratio and elongated root hairs, and depleted half of the investigated metabolites from soil extract. An important next step in the development of more field‐relevant EcoFABs will be the ability to include solid materials and microbial communities that reflect additional important aspects of soils.

## Author contributions

JS, JG and TRN developed the hypothesis, JS, JK, BJC, APK, JG, KL, KZ and BA conducted experiments, and JS, JK, BJC, APK, BA, PS, SK, BPB, KZ and DT performed data analyses. JS and TN wrote the paper, and JS, JK, BJC, APK, BA, PS, JG, KL, KZ, SK, BPB, DT, JV, AV, MW, JLD and TRN provided comments on the manuscript.

## Supporting information

Please note: Wiley Blackwell are not responsible for the content or functionality of any Supporting Information supplied by the authors. Any queries (other than missing material) should be directed to the *New Phytologist* Central Office.

**Fig. S1** Root morphology and exudate analysis capabilities of EcoFABs.
**Fig. S2** Total root length by laboratory.
**Fig. S3** Root : shoot ratio of EcoFAB‐grown *B. distachyon*.
**Fig. S4** Hierarchical clustering of root tissue and exudate metabolites.
**Fig. S5** Principal component analysis of soil extract exudate metabolites vs control.
**Fig. S6** Characteristic metabolites detected in exudates.
**Fig. S7** Metabolites reduced in exudates of soil extract grown plants by laboratory.
**Table S1** Participating laboratories and documented growth conditions for the reproducibility experiment.

**Table S2** Root tissue metabolite data.
**Table S3** Root exudate metabolite data.
