# Supplementary material for: Multilab EcoFAB study shows highly reproducible physiology and depletion of soil metabolites by a model grass
Source: New Phytol. 2019 Jan 24;222(2):1149–60. doi: 10.1111/nph.15662 (PMC6519027; doi:10.1111/nph.15662)
Supplement: Supplementary file 1 — Fig. S1 Root morphology and exudate analysis capabilities of EcoFABs. Fig. S2 Total root length by laboratory. Fig. S3 Root : shoot ratio of EcoFAB‐grown B. distachyon. Fig. S4 Hierarchical clustering of root tissue and exudate metabolites. Fig. S5 Principal component analysis of soil extract exudate metabolites vs control. Fig. S6 Characteristic metabolites detected in exudates. Fig. S7 Metabolites reduced in exudates of soil extract grown plants by laboratory. Table S1 Participating laboratories and documented growth conditions for the reproducibility experiment. [file NPH-222-1149-s001.pdf]

Multi-lab EcoFAB study shows highly reproducible physiology and depletion of soil metabolites by a model grass

Joelle Sasse[1,2], Josefine Kant[3], Benjamin J. Cole[1,2], Andrew P. Klein[4], Borjana Arsova[3], Pascal Schlaepfer[5], Jian Gao[1,2], Kyle Lewald[1,2], Kateryna Zhalnina[1,2], Suzanne Kosina[1,2], Benjamin P. Bowen[1,2], Daniel Treen[1,2], John Vogel[1,2], Axel Visel[1,2,6], Michelle Watt[3], Jeffery L. Dangl[4] & Trent R. Northen[1,2]

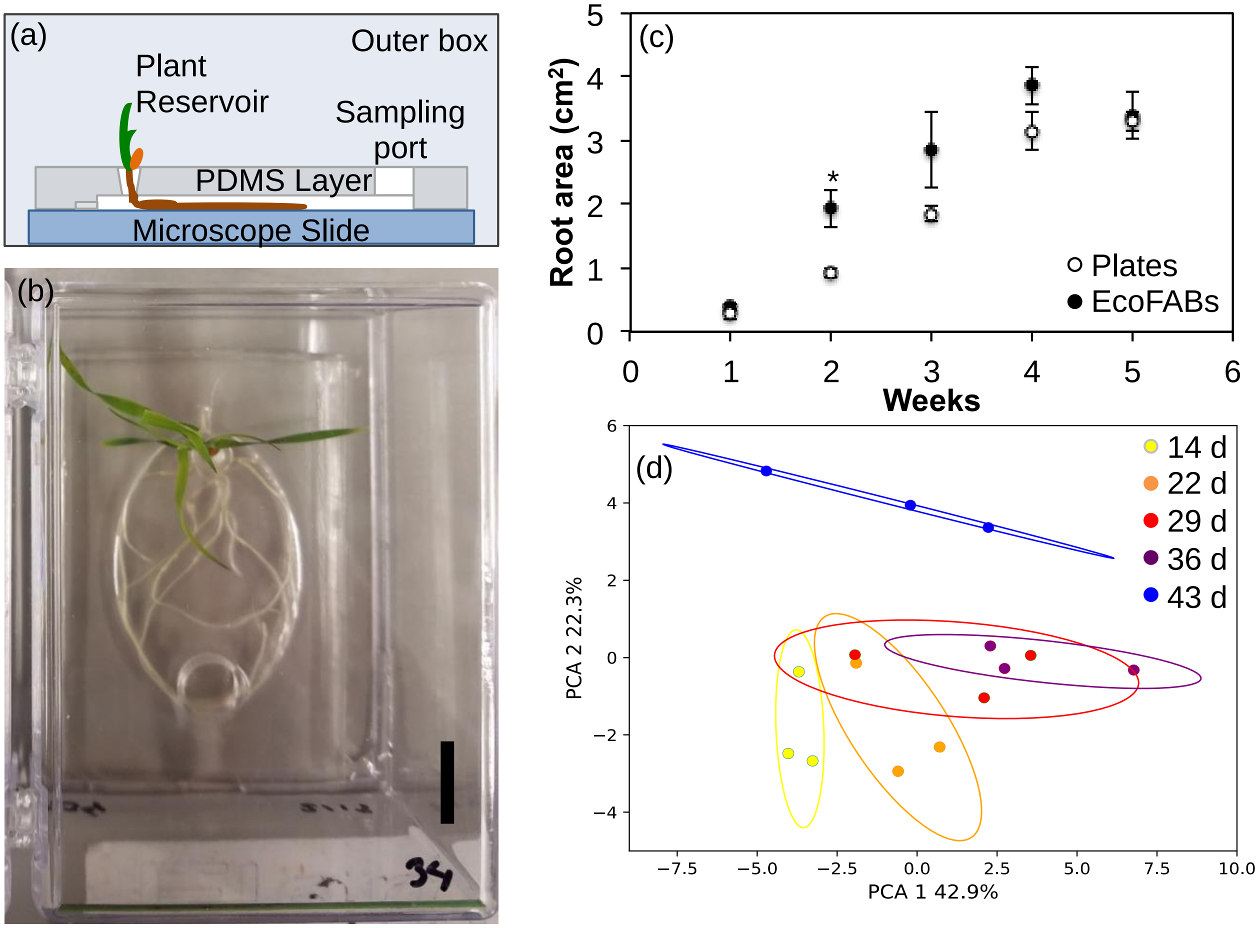

Fig. S1 Root morphology and exudate analysis capabilities of EcoFABs

(a) Conceptual design of EcoFAB, side view. (b) 21 dat *B. distachyon* growing in EcoFAB, top view. Scale bar: 1 cm. (c) Comparison of root area of *B. distachyon* grown on 0.5x MS plates (empty circles), and in EcoFAB containing 0.5x MS medium (full circles). Significant differences were detected only for the two week time point (data are means ± s.e.m., * = $p < 0.05$, n = 5). (d) Principal component analysis (PCA) of normalized peak heights of exudate metabolites from *B. distachyon* grown in EcoFABs for 14 d (yellow), 22 d (orange), 29 d (red), 36 d (purple), and 43 d (blue), n = 4. The data presented was generated by laboratory 1 independent of the reproducibility study presented.

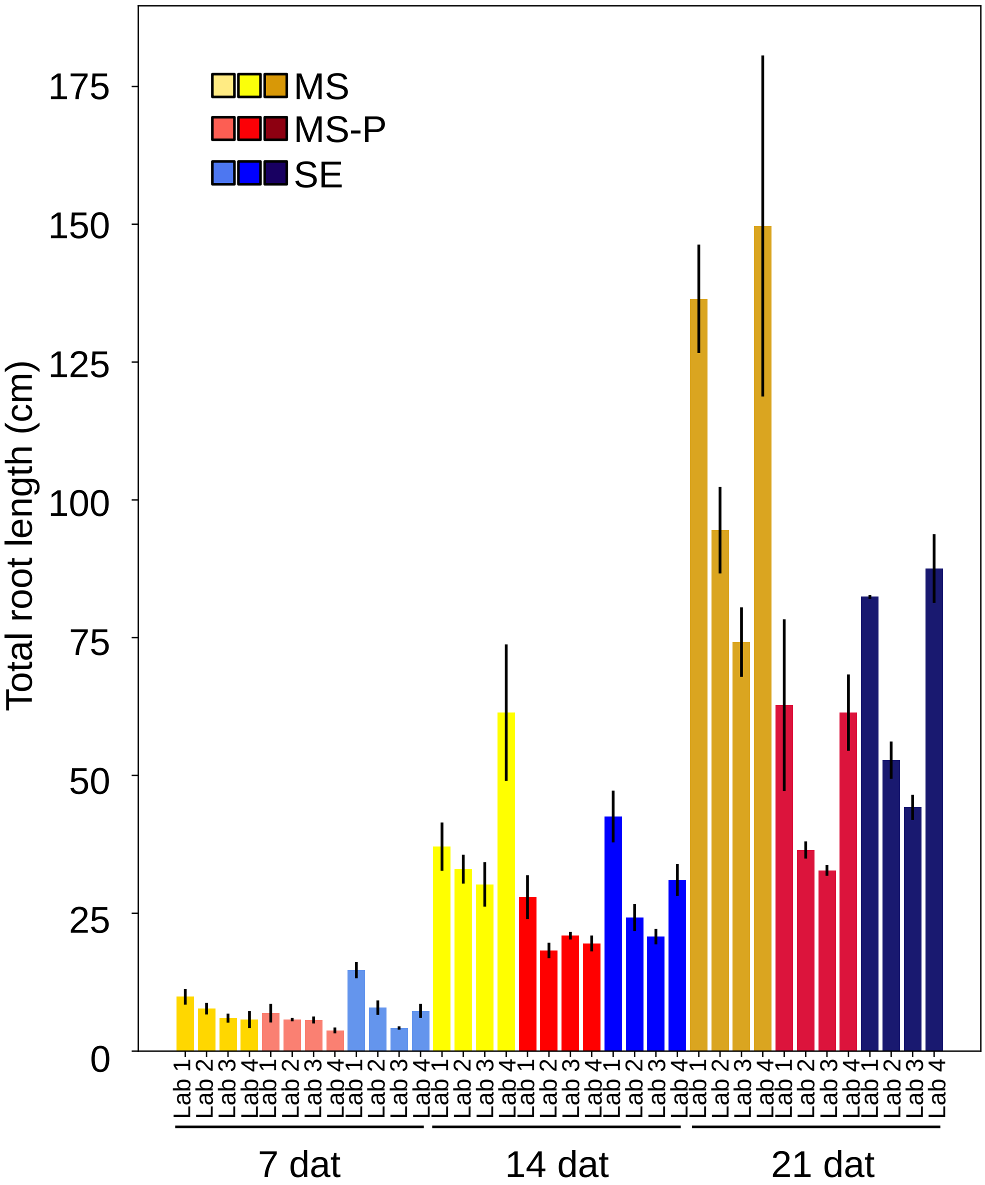

Fig. S2 Total root length by laboratory

Total root length in cm for *B. distachyon* 7, 14, and 21 days after transfer (dat) grown in 0.5x MS (MS), 0.5x MS-P (MS-P), or soil extract (SE). Values are means ± s.e.m. (n > 3). The same data is presented averaged across laboratories in Fig. 3.

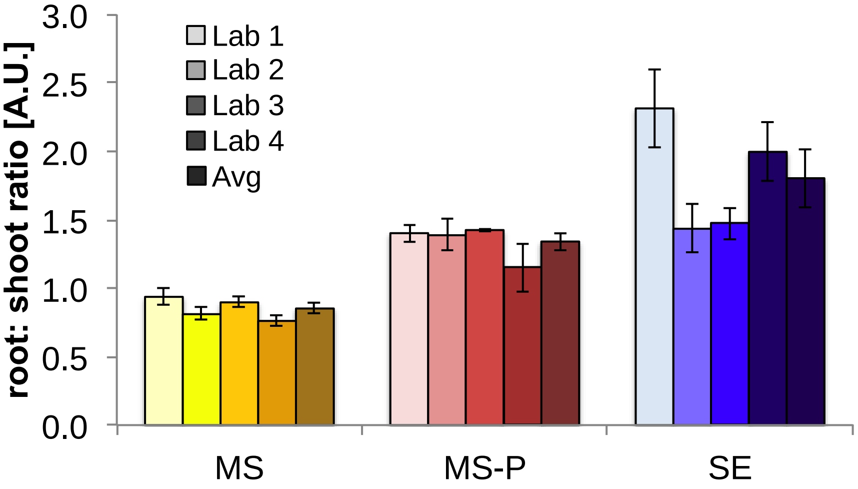

Fig. S3 Root:shoot ratio of EcoFAB-grown *B. distachyon*

B. distachyon was grown in 0.5x MS (MS, yellow), 0.5x-P (MS-P, red), and soil extract (SE, blue) for three weeks. Root and shoot fresh weight were determined (see Fig. 2a), and the root:shoot ratio was calculated for all participating laboratories (Lab1-4), as well as averaged across all laboratories (Avg). A.U.: arbitraty units. Values are means ± s.e.m. (n > 3).

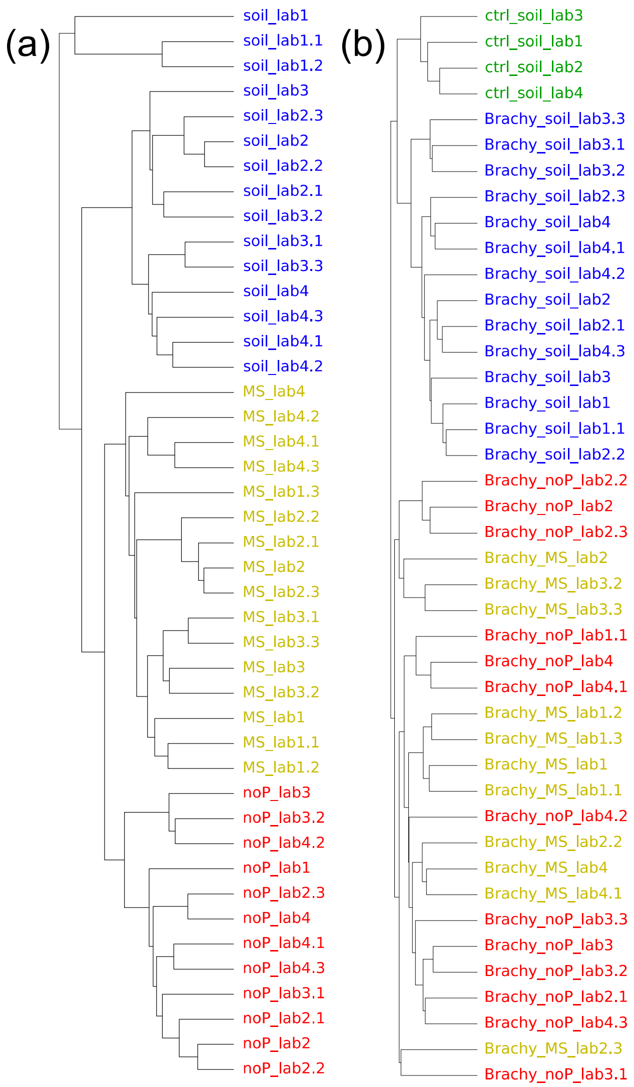

Fig. S4 Hierarchical clustering of root tissue and exudate metabolites

Bray Curtis hierarchical clustering of root tissue (a) and exudates (b) in 0.5x MS (yellow), 0.5x MS-P (red), soil extract (blue), and soil extract control (green). PCA plots of the same data are shown in Fig. 2.

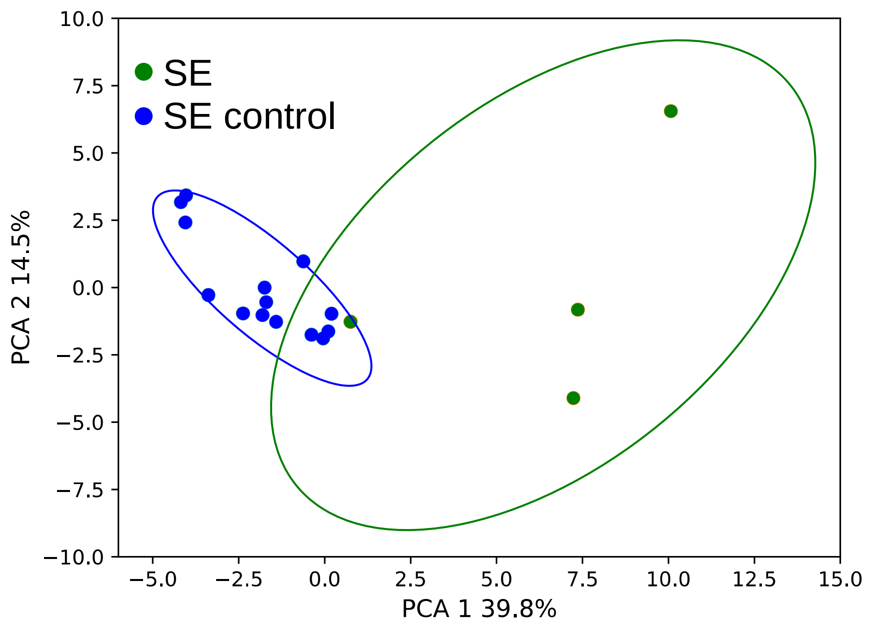

Fig. S5 Principal component analysis of soil extract exudate metabolites versus control

Principal component analysis of exudate metabolites of plants grown in soil extract (blue), and soil extract control (green).

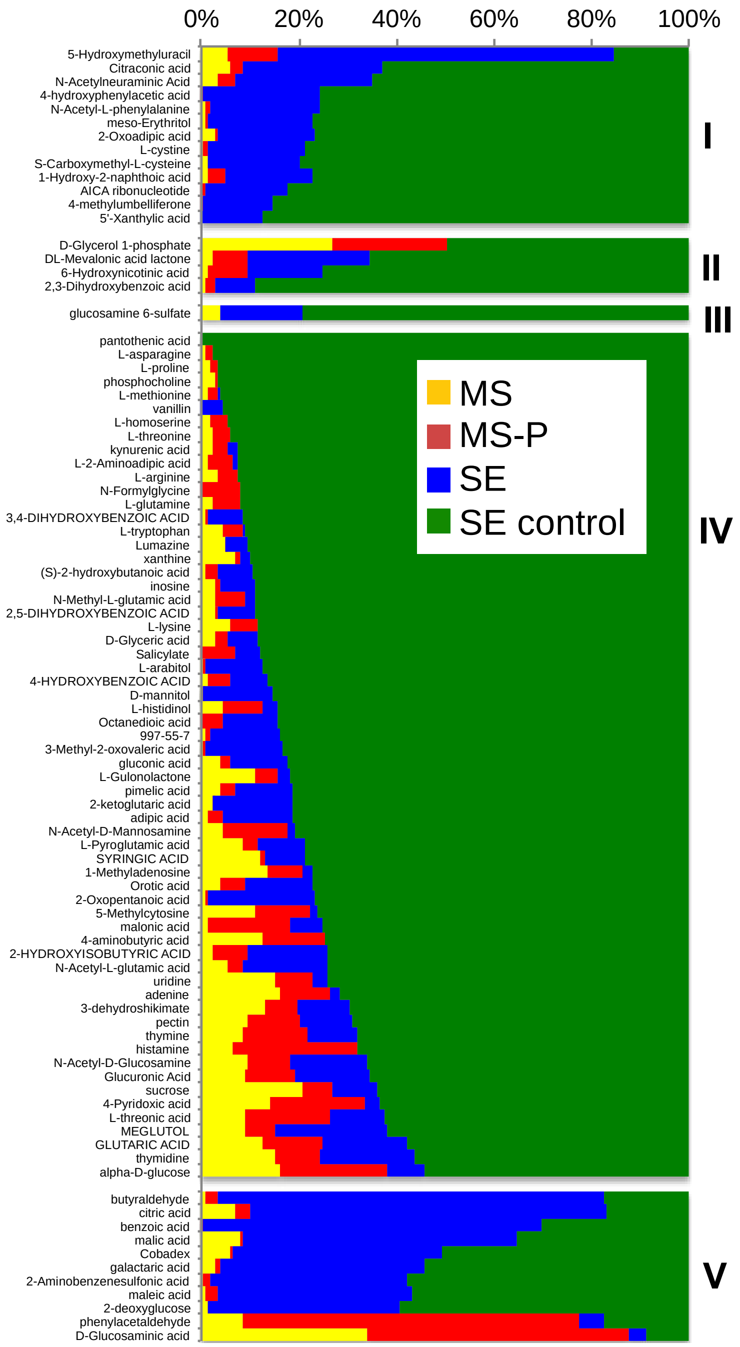

Fig. S6 Characteristic metabolites detected in exudates

Normalized relative peak height of metabolites differing between exudates of plants grown in 0.5x MS (MS, yellow), 0.5x MS –P (MS-P, red), soil extract (SE, blue), and soil extract controls (green) (Anova, $p < 0.05$). A selection of these metabolites is depicted in Fig. 5, and Fig. S6.

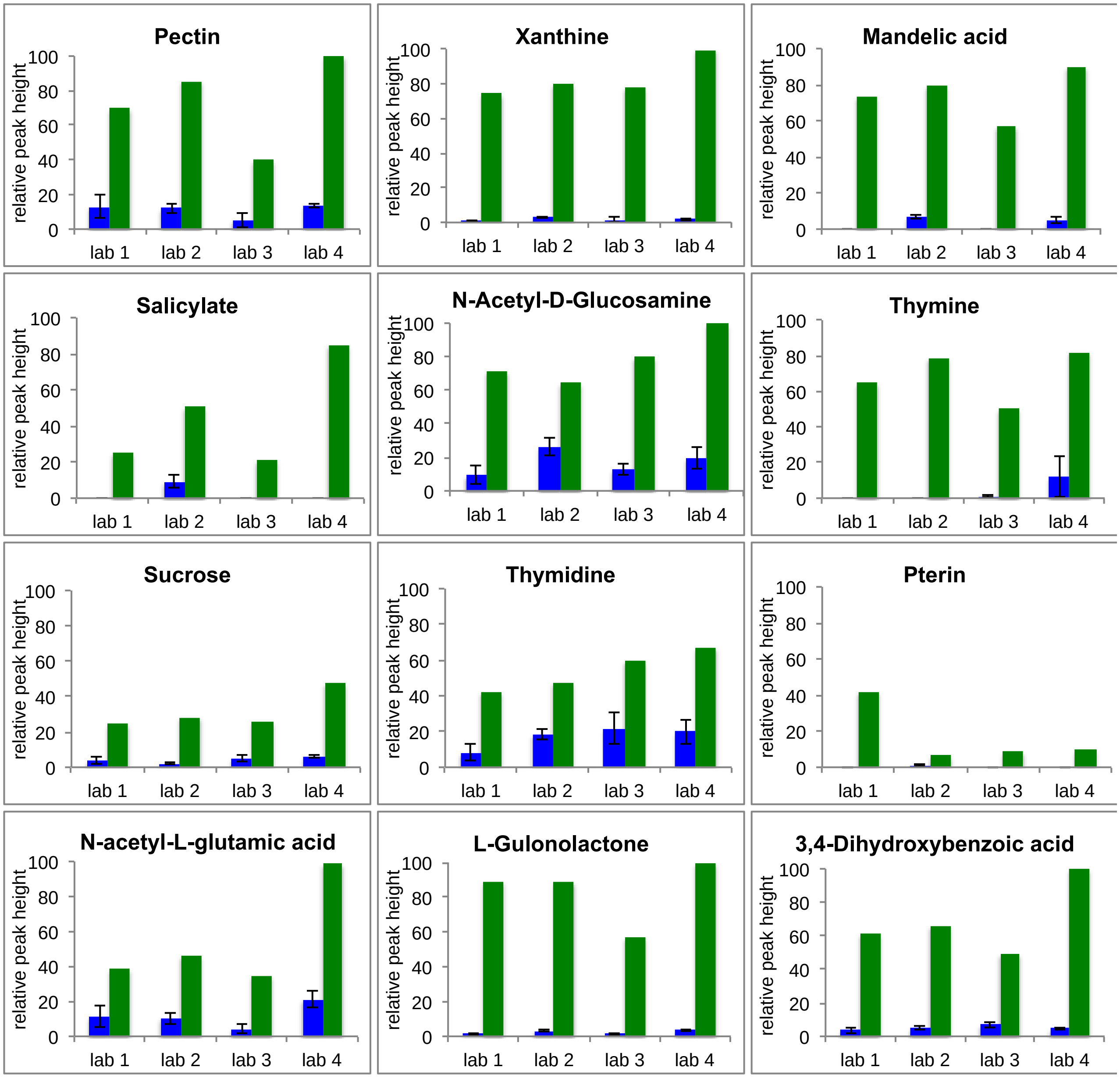

Fig. S7 Metabolites reduced in exudates of soil extract grown plants by laboratory

Relative peak height of selected metabolites differing between exudates of plants grown in soil extract (soil, blue), and soil extract controls (soil ctrl, green)(Anova, $p < 0.05$). Data are means ± s.e.m. (n=3 for soil, n=1 for control). Data averaged for all labs is presented in Fig. 5.

Table S1 Participating laboratories and documented growth conditions for the reproducibility experiment

Lab	Scientist[a]	Lab[a]	Location	day length (h)[b]	Temperature Day/night (˚C)[b]	light intensity (lux)[b]
lab 1	Joelle Schlapfer	Northen	Walnut Creek, CA, USA	16	26/25	5000
lab 2	Andrew Klein	Dangl	Chapel Hill, NC, USA	16	26/22	5200
lab 3	Borjana Arsova, Josefine Kant	Watt	Jülich, Germany	16	26/24	5300
lab 4	Ben Cole	Visel	Walnut Creek, CA, USA	16	23/22	5400

[a] the affiliations of scientists and their laboratories can be found in the author list
[b] as recorded on data loggers distributed to laboratories
